# The Citation Cloud of a biomedical article: a free, public, web-based tool enabling citation analysis

**DOI:** 10.5195/jmla.2022.1117

**Published:** 2022-01-01

**Authors:** Neil R. Smalheiser, Jodi Schneider, Vetle I. Torvik, Dean P. Fragnito, Eric E. Tirk

**Affiliations:** 1 neils@uic.edu, Professor in Psychiatry, University of Illinois at Chicago, Chicago, IL; 2 jodi@illinois.edu, Assistant Professor in iSchool, University of Illinois at Urbana-Champaign, Champaign, IL; 3 vtorvik@illinois.edu, Associate Professor in the iSchool, University of Illinois at Urbana-Champaign, Champaign, IL; 4 dean@xornet.com, Principal, Xornet, Inc; 5 etirk@xornet.com, Coprincipal, Xornet, Inc

**Keywords:** citation analysis, bibliometrics, information retrieval, evidence based medicine, science of science

## Abstract

**Background:**

An article's citations are useful for finding related articles that may not be readily found by keyword searches or textual similarity. Citation analysis is also important for analyzing scientific innovation and the structure of the biomedical literature. We wanted to facilitate citation analysis for the broad community by providing a user-friendly interface for accessing and analyzing citation data for biomedical articles.

**Case Presentation:**

We seeded the Citation Cloud dataset with over 465 million open access citations culled from six different sources: PubMed Central, Microsoft Academic Graph, ArnetMiner, Semantic Scholar, Open Citations, and the NIH iCite dataset. We implemented a free, public extension to PubMed that allows any user to visualize and analyze the entire citation cloud around any paper of interest A: the set of articles cited by A, those which cite A, those which are co-cited with A, and those which are bibliographically coupled to A.

**Conclusions:**

Citation Cloud greatly enables the study of citations by the scientific community, including relatively advanced analyses (co-citations and bibliographic coupling) that cannot be undertaken using other available tools. The tool can be accessed by running any PubMed query on the Anne O'Tate value-added search interface and clicking on the Citations button next to any retrieved article.

## BACKGROUND

Citation analysis is crucial for tracing the diffusion of knowledge across disciplines and over time, both at the micro level (individual citations) and macro level (global citation networks). For example, one may wish to follow citation chains (e.g., identifying the influence of a retracted article on later citing papers) [1,2]. Also, Hutchins et al. employed citation patterns to predict which articles are likely to contribute to the translation of basic studies into clinical advances [3], and Boyack and Klavans employed citations to identify research frontiers [4].

Citation analysis has largely been the province of scholars in the specialties of bibliometrics, scientometrics, and innovation and policy studies, who typically carry out extensive, time-consuming analysis of proprietary citation data licensed by commercial data providers. Many members of the scientific community may not take advantage of citation analysis to find relevant articles because it can involve the use of commercial databases for which they may not have access. Recently, iCite, an extensive set of open citations in the biomedical literature, was publicly released [5] with a monthly updated dataset (https://icite.od.nih.gov/). This provides a great opportunity for biomedical investigators and other interested parties, but, to date, there is no user-friendly interface for accessing or analyzing the citation data. Here, we describe Citation Cloud, a free, public extension to PubMed that allows any user to visualize and analyze the citation cloud around any article A: the set of articles cited by A, those which cite A, those which are co-cited with A, and those which are bibliographically coupled to A.

To say that an article B is co-cited with A means that they are both cited by the same article(s) C_i_ [6]. Co-citation is a measure of similarity not directly based on textual or topical similarity. Note that the co-citation relationship is not fixed but can vary over time depending on how many newer articles cite both A and B. According to Small, “It appears that an interpretation of the significance of strong co-citation links must rely both on the notion of subject similarity and on the association or co-occurrence of ideas […]. If it can be assumed that frequently cited papers represent the key concepts, methods, or experiments in a field, then co-citation patterns can be used to map out in great detail the relationship between these key ideas” [6].

In contrast, to say that an article B is bibliographically coupled to A means that they both cite some of the same articles C_i_ in their reference lists [7]. In other words, the reference lists of the two articles overlap, and the larger the number of shared references, the greater the degree of bibliographical coupling. This is also a measure of similarity that is not directly based on textual or topical similarity, although the fact that two bibliographically coupled papers share references suggests that they are also likely to share some methods, ideas, or topics. The bibliographically coupled relationship has the advantage that it can be calculated for any two articles regardless of when they were published. Also, this relationship is stable and will not change over time.

The new open access citations datasets can potentially enable a broad community of scientists to utilize citations in their studies of biomedical literature—not only the simple cites and cited by relationships but also the more sophisticated co-citation and bibliographically coupled relationships. Toward this end, we created a free, public, web-based tool for PubMed articles.

## CASE PRESENTATION

**Figure 1:**
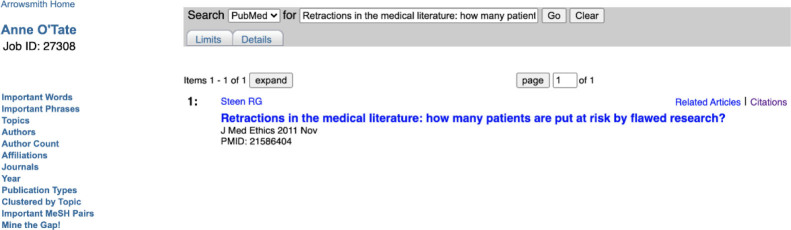
Screenshot of a PubMed query entered via the Anne O'Tate tool. Shown is the article retrieved using the title in the query box. The hyperlinked word Citations is displayed to the right of the article.

**Figure 2:**
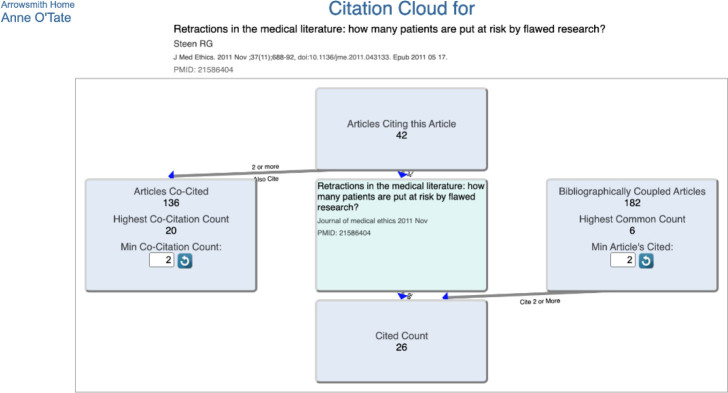
The citation cloud visualization for the article displayed in Figure 1.

The Anne O'Tate tool for searching PubMed was originally described in 2008 [8], with major additions described in 2021 [9], and provides a suite of tools that help summarize, mine, and drill down the results of a PubMed query. The Citation Cloud tool is an enhancement that provides four citation analyses for a record retrieved from PubMed using the Anne O'Tate tool. The Citation Cloud can be accessed by running any query on the Anne O'Tate value-added PubMed search interface (http://arrowsmith.psych.uic.edu/cgi-bin/arrowsmithuic/AnneOTate.cgi) [8,9] and clicking on the Citations button next to any retrieved article. For example, suppose we enter the query “Retractions in the medical literature: how many patients are put at risk by flawed research?” [1] to retrieve this single article (Figure 1). We then click on the Citations button next to the article and see its citation cloud visualization in a new tab (Figure 2).

The article of interest is in the center box, which is surrounded by four other boxes interlinked by arrows that show the direction of citations. Clicking on any box opens a new tab that shows the articles in that box and has hyperlinks to allow users to export the articles to PubMed or Anne O'Tate for further mining. The “Articles Citing this Article” box consists of all articles that cite the paper of interest. In this example, there are forty-two citing articles as of the date of this query. The “Cited Count” box consists of all articles in the reference list of the paper of interest. The “Articles Co-Cited” box consists of all articles that are cited by one or more papers in the “Articles Citing this Article” box. Highly cited papers may have a very large set of co-cited articles, so we allow users to adjust the co-citation count threshold as desired. Finally, the “Bibliographically Coupled Articles” box consists of all articles that cite papers in the reference list of the paper of interest.

**Figure 3:**
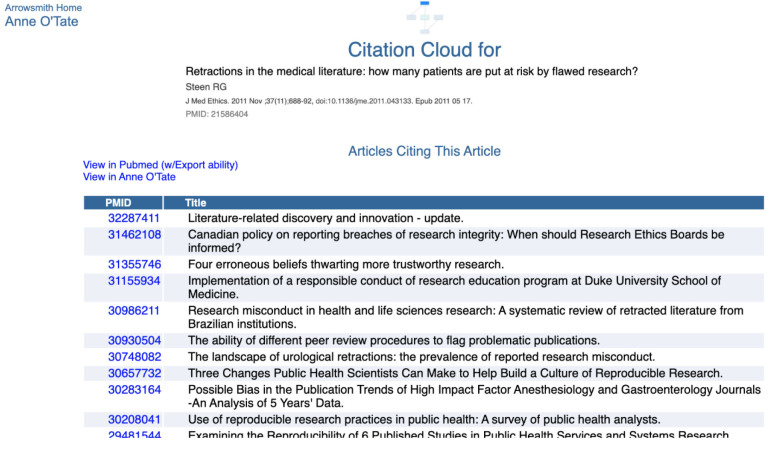
Screenshot of the contents of the “Articles Citing this Article” box. Only the top few records in the list are shown.

The upper box shows that forty-two articles have cited the article of interest; clicking on this box opens a new Results tab that lists the forty-two articles (Figure 3). Similarly, by clicking on the respective boxes, one can view and process articles that are cited by the article of interest, that are co-cited, or that are bibliographically coupled. The default option is to display a threshold of two, which means that at least two articles in the “Citing” box cited any article displayed in the “Co-Cited” box. Conversely, for the “Bibliographically Coupled” box, this means that each displayed bibliographically coupled article cited at least two references within the “Cited” box. The minimum threshold for display can be varied by the user in order to focus on the articles having the most similarity to the article of interest while minimizing the size of the list of articles displayed within the box.

Each box has two hyperlinks that permit the user to export the list to PubMed (which has the ability to export the citations in various formats) or to export the list to Anne O'Tate [8,9], where it can be further mined. For example, one can identify the most important words and phrases in the titles and abstracts of articles on the list as well as the most frequent topics, authors, or journals, [8,9].

As a rule, for any given article, there is little overlap between its set of citing articles, co-cited articles, bibliographically coupled articles, and PubMed Similar articles. Examining the full set of relationships provides insights that complement each other. If a given article of interest has just been published or has not been cited by another article, there are no citations or co-citations to analyze, yet one may still identify related articles using the PubMed Similar Articles function and the set of bibliographically coupled articles. Conversely, follow-up studies by the same team are likely to self-cite both the original paper and others by the team, so the set of co-cited articles is likely to relate to the team's broader interests.

**Figure 4:**
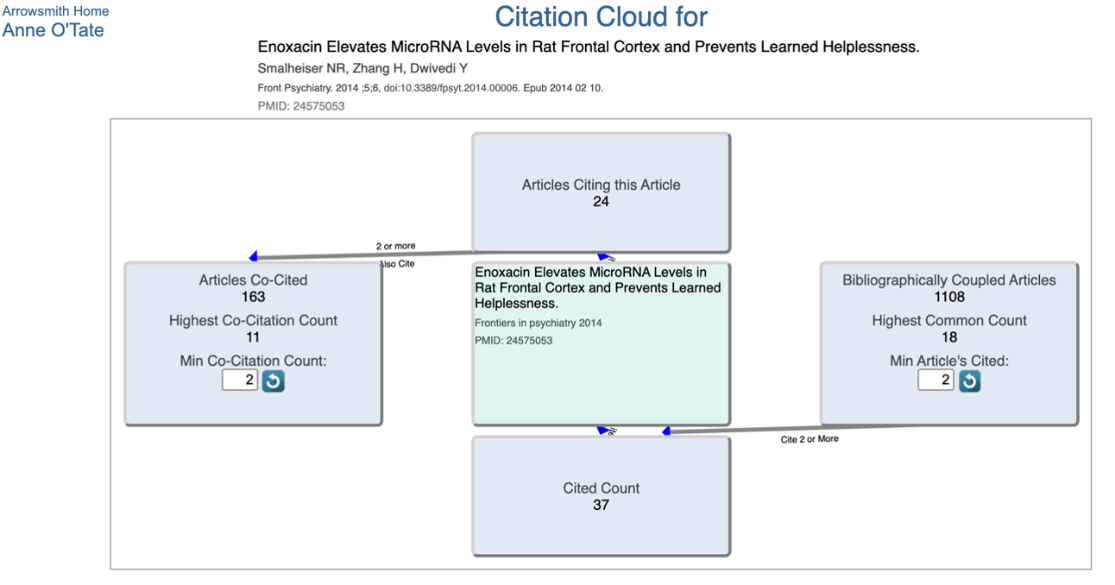
Screenshot of the Smalheiser et al. citation cloud.

**Figure 5:**
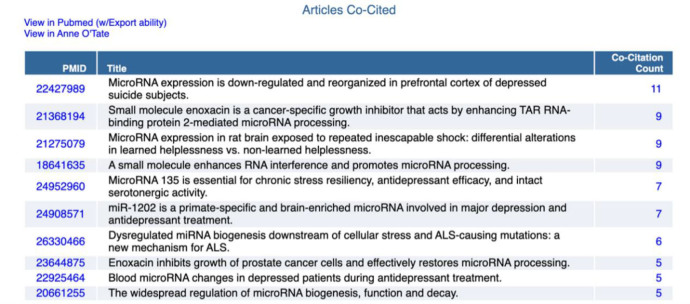
Top ten co-cited articles for the citation cloud shown in Figure 4.

**Figure 6:**
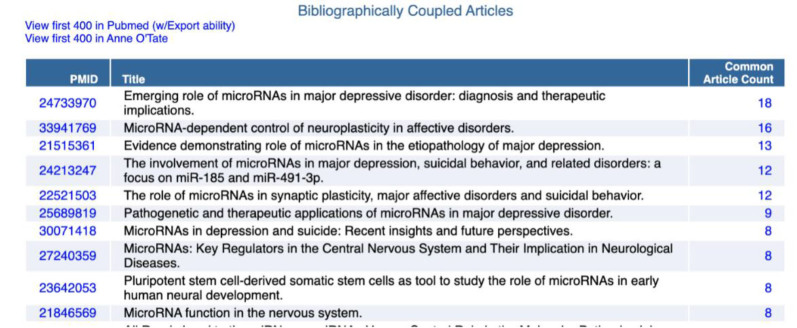
Top ten bibliographically coupled articles for the citation cloud shown in Figure 4.

When an article brings together two different lines of experimentation for the first time, the citation cloud may identify a mix of related articles across both lines. For example, an article by Smalheiser et al. titled “Enoxacin elevates microRNA levels in rat frontal cortex and prevents learned helplessness” [10] brings together studies of enoxacin as a regulator of microRNA production and studies of microRNA changes during animal models of depression and stress (Figure 4). The top ten co-cited articles include four that discuss enoxacin in various diseases and two other experimental studies by the same group (Figure 5). In contrast, the top ten bibliographically coupled articles all discuss microRNA function in depression and related disorders but show no overlap with the co-cited set (Figure 6). The top ten PubMed Similar Articles set shares two articles with the top ten co-cited set and none with the top ten bibliographically coupled set (not shown).

## DISCUSSION

Although we believe this tool is easy to use, users should be aware of several limitations. The initial seed dataset of open access citations (Appendix A) is static. The iCite dataset is updated monthly, and these new citations are automatically added to the Citation Cloud dataset. Any newly added PubMed Central citations that are not included in the iCite updates will also be added. However, since not all citations are captured by these sources [5] or are openly available [11], the set of citations is far from comprehensive. Whereas we incorporated citations from over seventeen-million unique articles indexed in PubMed, including proprietary citations from Web of Science and Scopus would have given access to over twenty-one-million articles. Another limitation is that the citation cloud surrounding a single article can be quite large, especially for review articles or citation classics. Thus, it may be too cumbersome to display a citation cloud to encompass an entire list of articles. Finally, the dataset and interface focus on PubMed articles rather than articles contained in other bibliographic databases.

We expect that this new tool will augment the power of the new open access citations datasets to enable a broad community of scientists to utilize citations in their studies of biomedical literature. The Citation Cloud tool may also be useful to biomedical investigators and public users who are not carrying out citation analysis per se. Co-cited and bibliographically coupled articles represent types of similarity that are complementary to the PubMed Similar Articles ranking [12] and thus may assist in increasing recall for information retrieval [13], such as finding relevant literature for systematic reviews [14].

## Data Availability

Information regarding the open citation dataset and the EAV architecture of our web tool are presented in Appendix A.
