# Supplementary material for: The Citation Cloud of a biomedical article: a free, public, web-based tool enabling citation analysis
Source: J Med Libr Assoc. 2022 Jan 1;110(1):103–8. doi: 10.5195/jmla.2022.1117 (PMC8830385; doi:10.5195/jmla.2022.1117)
Supplement: Supplementary file 1 — Appendix A [file jmla-110-1-103-s01.docx]

**Appendix A**

We seeded the Citation Cloud dataset with a very extensive set of open access citations which was culled from six different sources (Table 1), and included the initial release of the NIH iCite dataset in October 2019 [5].

**Table 1. Initial dataset of open citations to seed the Citation Cloud.**

**Source # of citation pairs**

PubMed Central 139,940,526

Microsoft Academic Graph 251,477,744

ArnetMiner 87,831,105

Semantic Scholar 199,871,665

Open Citations 175,634,784

iCite 418,767,235

**All combined 465,091,065**

The combined set represents the union of citation pairs (article A cites article B) for all six sources after removing duplicates. The article pairs comprise 17,681,409 unique PMIDs.

**EAV database architecture.**

An Entity-Attribute-Value (EAV) database structure was created to enable the generic storing and efficient querying of the sparsely populated PubMed source XML documents. It stores both single and multi-valued elements, naturally handles sparse data (not all documents contain all possible elements), and stores new elements (previously not found in a PubMed source document) without the need for database modification such as table creation, column creation, or index creation.

Traditional relational database structures use the technique of normalizing multi-valued elements into separate tables. There are positives and negatives to this. On the positive side, indexes may be created on the columns belonging to a multi-valued element. On the negative side, the normalizing process requires work to design the tables and design the indexes and requires custom coding to extract and populate the tables. Additionally, with sparse datasets such (i.e. PubMed) normalized models suffer from inefficiencies in disk usage and thus query performance, due to many empty or NULL values where no values exist in the source data (e.g. no FirstName in source but having a dedicated FirstName column in the database).

The EAV model as implemented combines the positives of indexing and none of the design and extract work. However it does require that relatively more complex SQL queries be written.

Some EAV systems store all data - regardless of type (string, number, date, etc) - as a string. This leads to problems when for instance, one is trying to sort by a number field but the values are stored as strings, in which case the query would sort incorrectly (e.g. 1, 12, 2, 200, 3…). Other EAV systems store different data types in specific columns that match the incoming data type. This is more efficient in terms of index use and query speed, but makes for even more complex queries and updates. Our EAV database structure incorporates a novel technique against two specific data types (strings and integers) that allows for simple storage to a single string column, yet makes use of indexes specific to those two data types. Future versions can be expanded to handle other data types such as Dates.

The technique automatically stored strings into a virtual column named ‘valshort’ which is indexed to the first 40 characters of the original string value. This allows one to quickly search against string values known to be short (e.g. LastName) by querying against the database’s fully indexed valshort column rather than against the non-indexable large Text column ‘val’. Integer values are likewise automatically converted by the database from the original XML string values and stored and indexed to the virtual ‘valint’ column. These virtual columns do not cause redundant storage space to be consumed as would real columns. They only consume storage within their given indexes.

Here is an example of a query which returns the list of co-citers within the Citation Cloud:

SELECT t1.aid as 'pmid', citer4.val as 'title'

FROM (

SELECT

citer.aid as aid

FROM aelement as citer

JOIN eir en2 ON

en2.hs='/PubmedArticle/PubmedData/ReferenceList/Reference/ArticleIdList/ArticleId'

WHERE citer.eirid=en2.eirid AND citer.valint=20072710

) as t1

LEFT JOIN eir en4 ON en4.hs='/PubmedArticle/MedlineCitation/Article/ArticleTitle'

LEFT JOIN aelement as citer4 ON citer4.aid=t1.aid and citer4.eirid=en4.eirid

ORDER BY pmid DESC

which returns (1st 10 shown):

+--------------+------------------------------------------------------------------------------------------------------+

| pmid | title |

+--------------+------------------------------------------------------------------------------------------------------+

| 30256792 | Self-citation is the hallmark of productive authors, of any gender. |

| 30197432 | Last Place? The Intersection of Ethnicity, Gender, and Race in Biomedical. |

| 28771391 | Gender Differences in Receipt of National Institutes of Health R01 Grants Among Junior Faculty at… |

| 28758138 | Author Name Disambiguation for PubMed. |

| 28509897 | Disambiguation of patent inventors and assignees using high-resolution geolocation data. |

| 28412964 | MeSH Now: automatic MeSH indexing at PubMed scale via learning to rank. |

| 27942200 | Quantifying Conceptual Novelty in the Biomedical Literature. |

| 27457939 | Kin of coauthorship in five decades of health science literature. |

| 27367860 | Author Disambiguation in PubMed: Evidence on the Precision and Recall of Author-ity among NIH-Funded |

| 27213780 | Two Similarity Metrics for Medical Subject Headings (MeSH): An Aid to Biomedical Text Mining and... |

...

Another example from the Citation Cloud returns a list of articles that are Bibliographically Coupled to pmid 20072710. The columns returned are pmid, title and common_article_count (the number of articles cited by the given pmid that are in common with the articles cited by the target pmid 20072710):

SELECT t1.aid_bca as 'pmid', caTitleEle.val as 'title', t1.bca_cac 'common_article_count' /* common article count */

FROM

(

SELECT ca.aid as 'aid_bca', COUNT(ca.valint) as 'bca_cac' /* common article count */

FROM aelement PARTITION (active) as target

JOIN eir en2 ON en2.hs='/PubmedArticle/PubmedData/ReferenceList/Reference/ArticleIdList/ArticleId'

/* join to other articles referencing same article as the target does */

JOIN aelement ca ON ca.eirid=en2.eirid AND ca.valint=target.valint AND ca.aid<>target.aid

WHERE target.eirid=en2.eirid AND target.aid=20072710

GROUP BY aid_bca

HAVING bca_cac>=".$MIN_bibc_count."

ORDER BY bca_cac DESC, aid_bca DESC

)

AS t1

/* join to title element of coupled article */

JOIN eir enTitle ON enTitle.hs='/PubmedArticle/MedlineCitation/Article/ArticleTitle'

LEFT JOIN aelement as caTitleEle ON caTitleEle.aid=aid_bca and caTitleEle.eirid=enTitle.eirid

which returns (1st 10 shown):

+----------+--------------------------------------------------------------------------------------------------------------+----------------------+

| pmid | title | common_article_count |

+----------+-------------------------------------------------------------------------------------------------------------------------------------+

| 29271976 | Gaps within the Biomedical Literature: Initial Characterization and Assessment of Strategies for Discovery. | 3 |

| 25661592 | Context-driven automatic subgraph creation for literature-based discovery. | 2 |

| 25472905 | Mammalian Argonaute-DNA binding? | 2 |

| 24376375 | Studying PubMed usages in the field for complex problem solving: Implications for tool design. | 2 |

| 23894639 | Has large-scale named-entity network analysis been resting on a flawed assumption? | 2 |

| 22195132 | SEACOIN--an investigative tool for biomedical informatics researchers. | 2 |

| 30533534 | Demystifying probabilistic linkage: Common myths and misconceptions. | 1 |

| 30294517 | Knowledge-based biomedical Data Science. | 1 |

| 30272675 | How user intelligence is improving PubMed. | 1 |

| 30266789 | Literature-based automated discovery of tumor suppressor p53 phosphorylation and inhibition by NEK2. | 1 |

...

In a non-EAV db configuration, we would have to create a secondary table to handle the many-to-many relationship sourced from PubMed’s /PubmedArticle/PubmedData/ReferenceList/Reference/ArticleIdList/ArticleId element, re-parse the entire corpus of PubMed just to get that one element, write dedicated code to deal with importing that element, and still have to JOIN to another table to get the Article Title to produce the above query results. With the EAV database structure, we had already imported every element from PubMed in a single generic routine so we already had the above element stored. No special tables or import routines were needed to deal with this (or any of the other multi-valued elements), and indexes were already created and optimized. Given these benefits, we anticipate greatly accelerated development time and little if any new database design and maintenance for future enhancements and projects.

The Architecture supporting The Citation Cloud is comprised of LINUX Ubuntu Server 18.04 LTS, Perl 5 version 26, and MySql 5.7.
